# Coronary artery fistula; coronary computed topography – The diagnostic modality of choice

**DOI:** 10.1186/1749-8090-3-41

**Published:** 2008-07-05

**Authors:** SA Early, TB Meany, HM Fenlon, J Hurley

**Affiliations:** 1 Department of Cardiothoracic Surgery, Mater Misericordiae University Hospital and Mater Private Hospital, Dublin, Ireland; 2 Department of Radiology, Mater Misericordiae University Hospital and Mater Private Hospital, Dublin, Ireland; 3 Department of Cardiology, Limerick Regional Hospital, Limerick, Ireland

## Abstract

Coronary artery fistulae (CAF) are rare anomalies. They are vascular communications between the coronary arteries and other cardiac structures, either cardiac chambers or great vessels. There can be considerable variation in the course of a coronary artery fistula. We report a case of a coronary artery fistula between the left circumflex coronary artery and the right and left atria. CAF are often diagnosed by coronary angiogram, however with the advent of new technologies such as Coronary Computed Tomography Angiography (Coronary CTA) the course and communications of these fistulae can be delineated non-invasively and with greater accuracy.

## Clinical summary

A 38-year-old woman presented with a 20-year history of chest pain and palpitations. Her past medical history was unremarkable. Clinical examination revealed a pansystolic murmur. A coronary angiogram was performed which identified a large fistula originating from the left circumflex coronary artery draining to the pulmonary artery. There was no evidence of any coronary artery disease. ECG, Echocardiography and Carotid Doppler's were normal. She subsequently underwent an ECG-gated contrast-enhanced coronary CT angiography study using a dual-tube 128 slice multidetector CT (Siemens, Erlangen Germany). This demonstrated a large fistula between the left circumflex artery and both the posterior aspect of the upper right atrium and the anterior wall of the left atrium.

**Figure 1:**
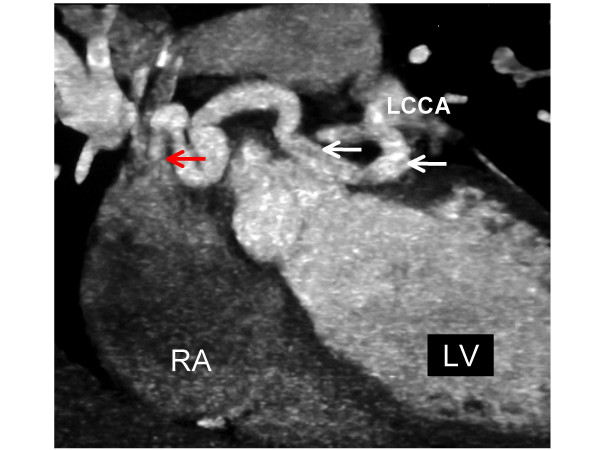
A coronal oblique image from an ECG-gated, contrast-enhanced CT coronary angiogram depicting a tortuous fistula (white arrows) originating from the left circumflex coronary artery (LCCA) and running in the atrio-ventricular groove to drain (red arrow) into the right atrium (RA).

**Figure 2:**
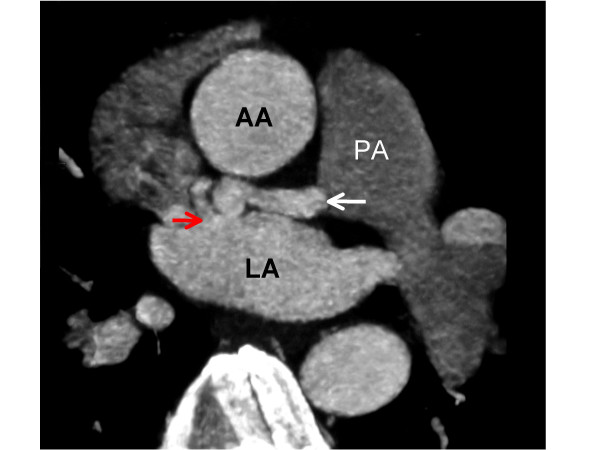
**An axial image from an ECG-gated, contrast-enhanced, coronary CT angiogram depicting the fistula (white arrow) running in the atrio-ventricular groove posterior to the ascending aorta (AA) and communicating (red arrow) with the left atrium (LA)**. Pulmonary artery (PA).

**Figure 3:**
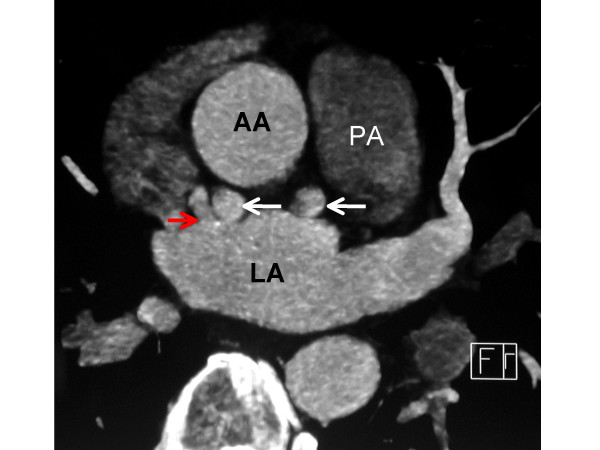
An axial image from an ECG-gated, contrast-enhanced coronary CT angiogram at a level just caudal to figure 2 demonstrates the tortuous nature of the fistula (white arrows) and depicts the fistula draining into the left atrium (LA).

The patient underwent surgical intervention. A median sternotomy was carried out and complete cardiopulmonary bypass was performed. Cardiac arrest was induced with cold crystalloid cardioplegia. The fistula was identified at the origin of the left circumflex artery running along the dome of the left atrium draining through the medial wall of the right atrium. There was also a small communication between the fistula and the left atrium. The fistula was traced back to its origin, at beginning of the circumflex artery where it was closed directly. The distal communications were also closed directly in both the right and left atria. The patient's postoperative course was uneventful and she was discharged home on the 10^th ^postoperative day. Her condition remains stable three months after the operation (see figures 1, 2 and 3).

## Discussion

Coronary artery fistulae are uncommon vascular communications between the coronary arteries and other cardiac structures. There are a limited number of cases described in the literature. It is reported that 0.1%–0.2% of all patients who undergo selective coronary angiography are diagnosed with a CAF [1]. CAF most commonly involve the right coronary artery (60%) but can involve both coronary arteries (5%) [2]. CAF can be congenital or acquired. Congenital CAF are thought to arise as a result of incomplete embryonic development; normally the coronary arteries communicate with the great vessels and chambers of the heart via sinusoids and during development these sinusoids transform into a normally calibrated capillary network. It has been postulated that incomplete closure of these sinusoids can result in CAF [3]. Acquired CAF can occur as a result of inflammation, atherosclerosis, and trauma or collagen vascular disease [4].

Previously the diagnosis of CAF has been made using conventional coronary angiography. With the advent of dual tube multidetector CT superior imaging can be now obtained using coronary CTA as described in this case. The patient's angiogram suggested that the fistula was draining into the pulmonary artery. However at the time of surgery the fistula was in fact draining into the right atrium and also communicating with the left atrium, which was clearly demonstrated in a non-invasive manner using coronary CTA.

Coronary CTA is a relatively new imaging modality that has been used for non-invasive coronary artery imaging since 2000 [5]. Prior to this earlier systems produced images that were of poor quality due to limitations with spatial and temporal resolution and image noise [6]. With the introduction of multi-detector computed tomography (MDCT) many problems with image quality have been overcome [7]. ECG-gated dualtube 128-slice MDCT is capable of producing high quality images with the ECG-gated image reconstruction algorithms allowing phase-correlated image data sets [8]. In this manner, Coronary CTA clearly delineates the cardiac chambers, the coronary arteries and coronary veins. It has been used as the technique of choice in demonstrating anomalous coronary artery anatomy and similarly is ideally suited to imaging patients with suspected coronary artery fistulae.

## Conclusion

This case highlights the benefit of and added information to be gained from the use of coronary CTA in the non-invasive diagnosis of coronary artery fistula. We recommend that coronary CTA should be obtained in all patients where a diagnosis of coronary artery fistula has been made at conventional angiography and that it should be regarded as the first test of choice in patients in whom the diagnosis is suspected.

## Competing interests

The authors declare that they have no competing interests.

## Authors' contributions

SE was responsible for data collection and drafting of the manuscript. TM was the referring physician and contributed to data interpretation. HF interpreted the radiological imaging, participated in the design of the figures and figure legends and revised the manuscript for important intellectual content. JH carried out the surgical procedure and was responsible for the surgical management of the patient. All authors read and approved the final manuscript.

## Funding

There was no funding obtained.
